# Clinical predictors of proteinuric remission following an LN flare - evidence from the UK JSLE cohort study

**DOI:** 10.1186/s12969-018-0230-4

**Published:** 2018-02-21

**Authors:** Eve M. D. Smith, Peng Yin, Andrea L. Jorgensen, Michael W. Beresford, on behalf of the UK JSLE Study Group

**Affiliations:** 1 Department of Women’s & Children’s Health, University of Liverpool, Institute In The Park, Alder Hey Children’s Hospital, East Prescott Road, Liverpool, L14 5AB UK; 2 Department of Paediatric Rheumatology, Alder Hey Children’s NHS Foundation Trust, Liverpool, UK; 3 Research Center for Biomedical Information Technology, Shenzhen Institutes of Advanced Technology, Chinese Academy of Sciences, Shenzhen, China; 4 Department of Biostatistics, Block F, Waterhouse Building, University of Liverpool, Liverpool, UK; 5 Department of Paediatric Rheumatology, Alder Hey Children’s NHS Foundation Trust, Liverpool, UK

**Keywords:** Juvenile-onset systemic lupus erythematosus, JSLE, Lupus nephritis, Active LN, Proteinuria, Prognosis

## Abstract

**Background:**

Proteinuria is a well-known risk factor for progression of renal dysfunction in a variety of chronic kidney diseases. In adult-onset Systemic Lupus Erytematosus (SLE) patients with lupus nephritis (LN), proteinuria takes a significant period of time to normalise, with proteinuric remission being associated with improved renal survival and reductions in mortality. The length of time required to attain proteinuric remission has not previously been investigated in Juvenile-onset SLE (JSLE). The aim of this study was to elucidate when proteinuric remission occurs, and whether clinical/demographic factors at LN onset bear influence on the time to proteinuric remission.

**Methods:**

Participants of the UK JSLE Cohort Study and Repository were included if they had active LN (renal biopsy and/or renal British Isles Lupus Assessment Grade (BILAG) score defined active LN) and proteinuria. Univariate Cox proportional hazard regression modelling was used to explore factors associated with time to proteinuric recovery. Covariates with *p*-value < 0.2 were included in a multivariable Cox regression model, and backward stepwise variable selection applied.

**Result:**

64/350 (18%) of UK JSLE Cohort Study patients fulfilled the study inclusion criteria. 25 (39%) achieved proteinuric remission within a median of 17 months (min 2.4, max 78). Within a multivariate Cox proportional hazard regression model, age at time of LN flare (*p = 0.007*, HR 1.384, CI 1.095–1.750), eGFR (*p = 0.035*, HR 1.016, CI 1.001–1.030) and haematological involvement (*p = 0.016*, HR 0.324, CI 0.129–0.812) at the time of LN onset were found to be significantly associated with time to proteinuric recovery.

**Conclusions:**

A significant proportion of children with LN have on-going proteinuria approximately two years after their initial flare. Poor prognostic factors all at time of LN onset include younger age, low eGFR, and concomitant haematological involvement.

## Background

Following a lupus nephritis (LN) flare, proteinuria has been shown to take a significant period of time to normalise in adults with systemic lupus erythematosus (SLE), with 53% of patients requiring up to 2 years to recover and only 74% recovering by 5 years [1]. Several adult SLE studies have shown that an early reduction in proteinuria following initiation of immunosuppressive therapy is associated with improved longer term renal outcomes [2–5]. Whilst proteinuria is on-going, differentiating between proteinuria due to on-going LN flare or chronic renal damage can be problematic. This leads the clinician to consider repetition of the renal biopsy, despite a lack of agreement as to the appropriate timing and indications for a repeat renal biopsy, particularly in children [6, 7].

Time to recovery from proteinuria in children with active LN receiving standard treatment has not been described to date. It is therefore of great interest to explore this within the national UK JSLE Cohort Study, to appreciate how long proteinuria persists for within a real world clinical setting (in contrast for example to a clinical trial setting). Identification of clinical and demographic factors at the onset of LN which are predictive of longer duration to resolution of proteinuria would be useful for stratifying patients as having high or low risk for achieving proteinuric remission, and helping to modify the intensity and duration of early immunosuppressive therapy.

## Methods

### Aim

The main aim of this study was to use data arising from the UK JSLE Cohort Study (a UK-wide multi-centre longitudinal cohort study collecting data as part of routine care) [8] between 1995 and 2015 to assess whether clinical and demographic factors can be used to predict time to proteinuric remission following an LN flare.

### Patients

Participants of the UK JSLE Cohort Study [8], aged < 16 years at the time of diagnosis and with ≥4 American College of Rheumatology (ACR) SLE classification criteria, were included in the current study if they had the following:

1. Active LN - defined in terms of having either renal biopsy defined active LN (International Society of Nephrology / Renal Pathology Society 2003 score, ISN/RPS classification score [9]), or renal British Isles Lupus Assessment Grade (BILAG) domain score of A or B [10].
2. Significant proteinuria - defined as a urine protein creatinine ratio (UPCR) or urine albumin creatinine ratio (UACR) of > 50 mg/mmol or a 24-h urine protein of ≥0.5 g.
3. At least two consecutive follow-up visits with significant proteinuria following its initial proteinuria onset.

Patients were therefore excluded if they did not have BILAG (renal domain score of C-E) or renal biopsy defined active LN, if their UPCR was ≤50 mg/mmol or 24-h urine protein was < 0.5 g, or if they had < two follow-up visits following the onset of proteinuria (as this precluded the ability for longitudinal follow-up). The proteinuria cutoff was chosen on the basis of the renal BILAG score, where a UPCR or UACR ratio of > 50 mg/mmol or a 24-h urine protein of ≥0.5 g is required for at least a score of B to be achieved, signifying moderate LN activity [10]. Further practical information on the BILAG score is detailed within a review by Lattanzi et al., comparing the BILAG score to other scores such as the SLEDAI score [11].

### Potential predictors of proteinuric recovery

Clinical and demographic factors (at the time of LN onset) were assessed as predictors within the analyses. Disease activity data was collected using the BILAG disease activity score at each routine clinic visit (approximately 3 monthly) [10]. Demographic details were collected using a standardized UK JSLE Cohort study case report form [8]. The demographic factors included gender, age at LN onset, ethnicity (caucasian/non-caucasian) and length of disease since diagnosis. Clinical renal factors consisted of proteinuria (spot UPCR or UACR), severe hypertension (blood pressure rising to > 170/110 mmHg within 1 month with grade 3 or 4 Keith-Wagener-Barker retinal changes), nephrotic syndrome (heavy proteinuria ≥3.5 g/day or protein-creatinine ratio of ≥350 mg/mmol or albumin-creatinine ratio of ≥350 mg/mmol, with hypoalbuminaemia and oedema), serum creatinine, presence of active urine sediment and estimated glomerular filtration rate (eGFR). Haematological features included haemoglobin, white cell count, neutrophils, lymphocytes and platelets. Immunological features consisted of complement factors 3 and 4 (C3/C4), anti-double-stranded DNA antibodies (anti-dsDNA), immunoglobulins (IgG, IgA, IgM), erythrocyte sedimentation rate (ESR) and c-reactive protein (CRP). Data on Coomb’s positivity was not included as the dataset was incomplete.

The physician global assessment, total numerical BILAG score and concomitant active involvement of different BILAG domains were also considered (score of A or B for a given organ domain) in the analyses, including constitutional, mucocutaneous, neuropsychiatric, musculoskeletal, cardiorespiratory, gastrointestinal, opthalmological and haematological domain involvement. Medication use at the time of LN onset was also recorded (hydroxychloroquine, azathioprine, mycophenelate mofetil, prednisolone, intra-venous immunoglobulin (IVIG), angiotensin inhibitor or angiotensin receptor blocker, previous rituximab or cyclophosphamide use) for the analyses.

### Study outcomes

Patients were categorised as having attained proteinuric recovery if their spot UPCR or UACR ratio was < 25 mg/mmol at two consecutive visits, or not recovered if the spot UPCR or UACR ratio was > 25 mg/mmol. The proteinuria cut off levels were chosen on the basis of the renal BILAG score as a spot UPCR or UACR ratio of < 25 mg/mmol would lead to a renal BILAG score of D, signifying inactive LN but previous renal involvement.

### Statistical analysis

The study was undertaken using retrospective data from a longitudinal patient cohort. The outcome for each patient was defined as time to the date of proteinuric recovery, or to the date of the last visit date if they had not achieved proteinuric recovery and were censored. Cox proportional hazard regression modelling was used to univariately test the association between each clinical/demographic variable of interest and outcome. Variables with missing data were tested with complete cases only univariately. Covariates with *p < 0.2* on univariate analysis were included in a multiple Cox regression model. Where > 10% of the data was missing for an included covariate, ‘MICE’ package in R version 3.2.0 was used to undertake multiple imputation [12]. Covariates to be retained in the final model were chosen by using a backward stepwise model selection procedure (threshold *p < 0.05*). Hazard ratios (HRs), 95% confidence intervals (CIs) and *p-values* were summarised for covariates present in the final model. The results were displayed graphically with Kaplan-Meier curves and risk tables. All analysis was undertaken with R version 3.2.0 [13].

## Results

**Fig. 1:**
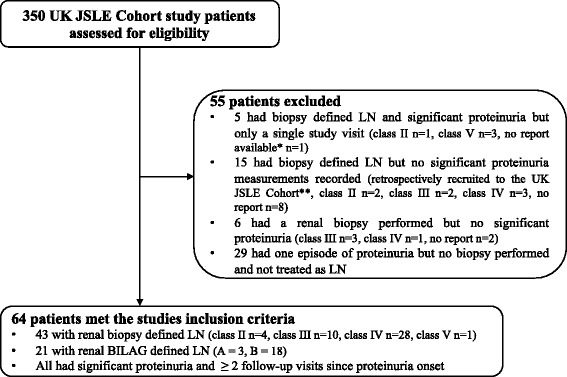
Participant flow diagram. ISN/RPS classification reported for renal biopsies. *Box ticked on the BILAG case report form that the patient had ‘histological evidence of active nephritis within 3 months of the clinic visit’ but the full biopsy report was not provided. **The UK JSLE Cohort Study largely recruits and prospectively collects clinical data from participants across the UK. At the time of initial study set up, patients seen between 1995-2006 were retrospectively recruited and limited retrospective clinical data collected

The study cohort consisted of 350 UK JSLE Cohort Study patients, of which 64/350 (18%) met the study’s inclusion criteria and were therefore considered within these analyses. 43/64 (67%) had renal biopsy defined LN (class II *n* = 4, class III *n* = 10, class IV *n* = 28, class V n = 1) and 21/64 (33%) had renal BILAG defined LN (A = 3, B = 18). 55/350 patients met some but not all of the inclusion criteria and were therefore excluded (see Fig. 1 for further details). 54/64 patients had LN at the time of their initial diagnosis and 10/64 had a subsequent recurrent episode of LN. During the study follow-up period, proteinuric remission was achieved in 25/64 (39%) patients, within a median of 17 months (interquartile range, IQR 3.5–49.2).

### Clinical / demographic factors influencing time to proteinuric recovery?

Using univariate Cox proportional hazard regression modelling, age at LN onset, serum creatinine, eGFR, neutrophil count, physician global assessment and BILAG defined haematological involvement all gave *p values* of *< 0.2* (see Table 1), and therefore were considered within a multiple Cox regression model. There was no statistical difference in the distribution of renal biopsy subclasses, or whether patients had LN at diagnosis or recurrent LN, amongst the recovered and not-recovered groups (*p = 0.388* and *p = 0.0784* respectively).

**Table 1:** Univariate Cox proportional hazard regression modelling looking at time to proteinuric remission

	Clinical and demographic factors at LN onset	Not recovered (*n* = 39)^a^	Recovered (*n* = 25)^b^	Hazard Ratio (95% CI)	*p-value*
Factors included within the multivariate model	Age at LN onset (yrs)	13.9 (6.4, 17.7)	13.6 (8.1, 17.9)	1.0007 (1.0001, 1.0013)	*0.013*
	S creatinine (micromol/l, NA = 8)	61 (34, 234)	50 (36, 177)	0.991 (0.976, 1.005)	*0.184*
	eGFR (ml/min/m^2,^ NA = 2)	104 (29, 159)	121 (29,153)	1.014 (0.999, 1.028)	*0.060*
	Neutrophil count (× 10^9^/L, NA = 2)	3.4 (1.1, 17.8)	3.44 (0.4, 12.33)	0.932 (0.837, 1.037)	*0.197*
	Physicians global assessment	23 (0, 75)	41 (1, 71)	1.027 (0.994, 1.061)	*0.107*
	Haematological involvement^c^	Y: 33, N: 6	Y: 13, N: 12	0.421 (0.187, 0.952)	*0.038*
Demographics, clinical features and laboratory investigations	LN ISN/RPS renal biopsy class^d^	Class II: 1 Class III: 5 Class IV: 15 Class V: 2	Class II: 3 Class III: 5 Class IV: 10 Class V: 2	0.769 (0.429, 1.378)	*0.388*
	LN at diagnosis or recurrent LN	Diagnosis: 33 Recurrent: 6	Diagnosis: 21 Recurrent: 4	0.320 (0.090, 1.138)	*0.078*
	Female gender	32/39 (82%)	18/25 (72%)	1.572 (0.642, 3.852)	*0.322*
	Caucasian ethnicity^e^	13/39 (33%)	13/25 (52%)	1.404 (0.637, 3.091)	*0.421*
	Length of disease at LN onset (days)	225 (0, 4857)	27 (0, 2679)	0.9999 (0.9994, 1.0005)	*0.934*
	Baseline Proteinuria^f^	149 (50, 2772)	252 (51, 1418)	0.9999 (0.9993, 1.0006)	*0.989*
	Severe hypertension (NA = 3)^g^	Y: 6, N: 31	Y: 3, N: 21	0.482 (0.140, 1.671)	*0.253*
	Nephrotic syndrome (NA = 3)^h^	Y: 7, N: 30	Y: 5, N: 19	0.853 (0.318, 2.342)	*0.765*
	Active urinary sediment (NA = 40)^i^	Y: 5, N: 9	Y: 3, N: 7	1.722 (0.402, 7.423)	*0.466*
	Haemoglobin (g/dl)	10.8 (5.6, 96)	11.3 (7.1, 14.9)	0.999 (0.949, 1.052)	*0.979*
	WCC (×10^9^/L)	4.8 (2.5, 22.4)	6.4 (0.5, 9.1)	0.965 (0.885, 1.053)	*0.426*
	Lymphocytes (×10^9^/L, NA = 2)	1.40 (0.1, 5.0)	1.53 (0.1, 5.42)	0.970 (0.670, 1.410)	*0.879*
	Platelets (×10^9^/L)	245 (77, 589)	225 (82, 522)	0.998 (0.995, 1.002)	*0.342*
	ESR (mm/h, NA = 11)	40 (2, 170)	37.5 (4, 102)	0.994 (0.982, 1.006)	*0.353*
	CRP (mg/L, NA = 27)	5 (1, 19)	5 (1, 295)	0.998 (0.991, 1.005)	*0.601*
	C3 (g/L, NA = 7)	0.51 (0.18, 1.61)	0.71 (0.22, 1.31)	1.011 (0.301, 3.400)	*0.986*
	C4 (g/L, NA = 7)	0.06 (0.01, 0.90)	0.07 (0.02, 0.21)	0.282 (0.005, 15.171)	*0.537*
	Anti-dsDNA titres (IU/L, NA = 22)	119 (0, 3503)	220 (42, 3770)	0.999 (0.999, 1.000)	*0.577*
	IgG (g/L, NA = 27)	14.6 (0.9, 70.2)	11.8 (2.8, 33.1)	0.957 (0.895, 1.022)	*0.296*
	IgA (g/L, NA = 28)	2.06 (0.8, 4.9)	2.36 (0.3, 3.7)	0.870 (0.493, 1.542)	*0.636*
	IgM (g/L, NA = 28)	1.11 (0.4, 9.6)	(0.07, 2.5)	0.614 (0.271, 1.412)	*0.252*
Medications at LN onset	Hydroxychloroquine^j^	Y: 21, N: 18	Y: 12, N: 13	1.514 (0.663, 3.411)	*0.328*
	Azathioprine	Y: 8, N: 31	Y: 2, N: 23	0.520 (0.123, 2.222)	*0.377*
	Mycophenolate Mofetil	Y: 10, N: 29	Y: 6, N: 19	1.400 (0.550, 3.590)	*0.475*
	Prednisolone	Y: 24, N: 15	Y: 14, N: 11	1.260 (0.560, 2.860)	*0.581*
	Intravenous immunoglobulin	Y: 2, N: 37	Y: 2, N: 23	0.790 (0.170, 3.640)	*0.762*
	Rituximab ever	Y: 2, N: 37	Y: 1, N: 14	0.373 (0.046, 2.982)	*0.351*
	Cyclophosphamide ever	Y: 3, N: 36	Y: 2, N: 23	0.574 (0.132, 2.563)	*0.463*
	ACEi or AT2i^k^	Y: 11, N: 28	Y: 4, N: 21	0.753 (0.263, 2.222)	*0.607*
Concomitant BILAG defined organ involvement	Constitutional involvement	Y: 15, N: 24	Y: 15, N: 10	1.410 (0.621, 3.183)	*0.411*
	Mucocutaneous involvement	Y: 23, N: 16	Y: 15, N: 10	0.971 (0.431, 2.182)	*0.936*
	Neuropsychiatric involvement	Y: 3, N:36	Y: 3, N: 22	0.920 (0.261, 3.231)	*0.901*
	Musculoskeletal involvement	Y: 19, N: 20	Y: 14, N: 11	0.612 (0.251, 1.473)	*0.272*
	Cardiorespiratory involvement	Y: 3, N: 36	Y: 5, N: 20	1.253 (0.462, 3.422)	*0.663*
	Gastrointestinal involvement	Y: 0, N: 39	Y: 3, N: 22	1.311 (0.361, 4.761)	*0.680*
	Ophthalmological involvement	Y: 0, N: 39	Y: 2, N: 23	1.082 (0.221, 5.300)	*0.919*
	Total numerical BILAG score	11 (3, 27)	11 (1, 53)	0.980 (0.951, 1.021)	*0.424*

Summary statistics used for continuous variables in the recovered and not recovered columns (median, min, max), whereas number count was detailed for discrete variables. Missing data shown in brackets with NA. *p-values* are from univariate Cox Proportion hazard models. ^a^Not recovered = not attained proteinuric recovery during the follow up period. ^b^Recovered = attained proteinuric recovery defined as if their spot UPCR or UACR ratio being <25mg/mmol at two consecutive visits. ^c^BILAG defined active organ domain involvement (score of A or B). ^d^Active LN defined in terms of having either renal biopsy defined active LN (ISN/RPS score), 32 or renal BILAG domain score of A or B. 43/64 patients had renal biopsy defined LN and 21/64 had renal BILAG defined LN. ^e^Patients grouped as caucasian / non-caucasian for the purposes of the analysis. ^f^Baseline Proteinuria = spot UPAC or UAUC measurements depending on hospital laboratory. ^g^BILAG defined severe hypertension. ^h^Nephrotic syndrome = heavy proteinuria (> 50 mg/kg/day or > 3.5 g/day or protein-creatinine ratio > 350 mg/mmol or albumin-creatinine ratio > 350mg/mmol) + hypoalbuminaemia + oedema. ^i^Active urine sediment = pyuria (> 5 WCC/hpf), haematuria (> 5 RBC/hpf) or red cell casts in absence of other causes. ^j^Medication use (yes) or non-use (no) considered rather than absolute drug dose. ^k^ACEi or AT2i = Angiotensin inhibitor or angiotensin receptor blocker

After applying stepwise backward variable selection to the multivariable Cox regression model, it was identified that those who were older, or had higher eGFR values at the time of LN onset, were more likely to achieve proteinuric recovery. Further, those with haematological involvement at the time of LN onset were less likely to achieve proteinuric recovery (see Table 2).

**Table 2:** Multivariable–Cox regression model displaying factors associated with time to proteinuric recovery

Clinical and demographic factors at LN onset	HR (95% CI)	*p value*
Age at LN onset (years)	1.384 (1.095, 1.750)	*0.007*
eGFR	1.016 (1.001, 1.030)	*0.035*
Haematological involvement	0.324 (0.129, 0.812)	*0.016*

*eGFR* estimated glomerular filtration rate, *HR* hazard ration, *CI* confidence interval

Multivariable Cox regression model after applying variable selection

**Fig. 2:**
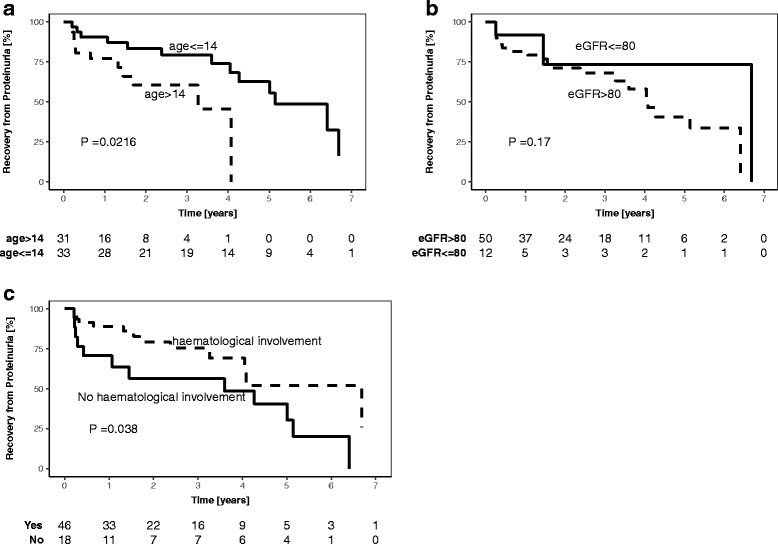
legend: Kaplan-Meier plot for age, eGFR and haematological involvement. **a** Patients aged <=14 years, *n* = 33. Patients aged > 14 years, *n* = 31. **b** Patients with eGFR of >80mls/min, *n* = 50. Patients with eGFR<=80mls/min, *n* = 12. The Kaplan-Meier plot for eGFR appeared to marginally deviate from the assumption of proportional hazards, however this could be due to the very small number of patients in the <80mls/min group after the second time-point. **c** Patients with BILAG defined haematological involvement, *n* = 46. Patients without haematological involvement, *n* = 18. Non-imputed data used for development of Kaplan-Meier plots, therefore *n* = 64 for age plot, 62 for eGFR and 64 for BILAG defined haematological involvement plot. The table below each plot shows the number of patients who continue to be at risk of developing LN at each time point. *P*-values on the Kaplan-Meier plots are from log-rank tests of dichotomised variables, and therefore differ from the regression model *p*-values shown in Table 2

Kaplan Meier plots for age, eGFR and BILAG defined haematological involvement are shown in Fig. 2. Figure 2a demonstrates that at a given time, patients who are older at the time of LN onset (> 14 years vs < 14 years) are more likely to achieve proteinuric recovery. The median patient age across the whole patient group was 14 years, therefore, that is why this cut-off was used to divide patients into younger or older groups. Median time to recovery was 4.95 years (95% CI: 0.33–9.09) in the younger age group and 0.65 years (95% CI: 0.29–5.31) in the older age group (*p = 0.021)*. The Kaplan Meier plot for eGFR (Fig. 2b) divides patients into two clinically relevant sub-groups with an eGFR of less or greater that 80 mls/min, demonstrating that at a given time, patients with a eGFR of > 80 mls/min at LN onset are more likely to recover. Those with eGFR levels < 80 mls/min had a median time to recovery of 2.00 years (95% CI: 0.43–8.85) whilst those above 80 mls/min had median time to recovery of 1.82 years (95% CI: 0.28–7.95, *p = 0.170*). Figure 2c illustrates that at a given time, patients without haematological involvement at LN onset are more likely to attain proteinuric recovery at each time point. Median time to recovery was 1.73 years (95% CI: 0.28–8.34) in those without involvement and 2.14 years (95% CI: 0.30–8.13) in those with haematological involvement (*p = 0.038*).

## Discussion

Using data from a large national cohort of patients recruited to the UK JSLE Cohort Study, this study has demonstrated that proteinuria can be persistent following a LN flare. Clinical and demographic data have been used within this study to characterise patients who are at increased risk of having a prolonged period of proteinuria following an LN flare. Early reduction in proteinuria following initiation of immunosuppressive therapy has been shown to be associated with improved longer term renal outcomes [1, 14] and patient survival [4] in adult SLE, therefore, appreciation of those who are likely to need longer to achieve proteinuric recovery may influence monitoring and treatment decisions. Out-with a LN setting, levels of albuminuria have been shown to relate to all cause and cardiovascular mortality [15, 16], with proteinuria reduction being the main target for halting the progression of diabetic [17] and many non-diabetic kidney diseases [18].

A total of 39% of patients were shown to reach proteinuric remission following a LN flare during the study period, within a median of 17 months (IQR 3.5–49.2). This observation provides useful information on the length of time necessary to achieve proteinuric recovery following a LN flare within real world clinical practice, as opposed to a tightly regulated clinical trial setting. A similar study in adult SLE showed proteinuric recovery to occur in 53% of patients by 2 years [1], suggest that proteinuria may take longer to normalize in children than adults with SLE. In a Korean study including 193 adults with SLE and severe proliferative LN, proteinuric remission was attained in 8% of patients within 12 months, 19% between 12 and 60 months, and 16% after more than 60 months post renal biopsy, suggesting that those with the most severe LN histological sub-types may need even longer to achieve proteinuric remission [14].

Younger age at the time of LN onset was shown to be the strongest predictor of having a prolonged time to proteinuric recovery, likely reflecting the more severe disease phenotype and potential genetic predisposition to JSLE/LN seen in younger patients [8, 19–24]. This study did not collect information on pubertal status, but in future work it would be useful to assess the influence of pubertal status on time to proteinuric recovery. Reductions in eGFR usually occur following significant renal damage and may be preceded by a period of hyper-filtration [25–27]. It is therefore not unexpected that low eGFR at the time of LN onset was associated with longer time to proteinuric recovery in the current study.

Haematological involvement at the time of LN onset was associated with a longer time to proteinuric recovery. It is of interest that individual haematological measures (e.g. haemoglobin, white count, platelets) were not individually associated with time to proteinuric recovery, but as reflected by the haematological domain of the BILAG score, combinations of abnormalities were found to be important. It can be speculated that concomitant haematological involvement may also affect the ability to intensify immunosuppressant treatment due to concerns about treatment toxicity, thereby influencing time to proteinuric recovery. Awareness of these factors is important for stratification of patients at the time of LN onset, and considering the intensity and duration of early immunosuppressive therapy. In future work it would also be of interest to look at whether patients were Coombs’ positive and their reticulocyte counts.

The strengths of this study lie in the large real-world patient cohort receiving a multitude of treatment regimens, as opposed to a tightly regulated clinical trial. Certain limitations of this study do however warrant recognition and should be addressed in future work. The study did not include all LN patients; with five biopsy proven LN patients excluded as they only had a single follow-up visit, and a further 15 patients excluded who were retrospectively recruited to the cohort and had insufficient proteinuria measurements recorded for meaningful analysis to be undertaken. The analysis looked at treatment at baseline and did not look at the influence of on-going treatment (steroids and DMARDs) due to the number and complexity of the different treatment plans used, precluding meaningful analysis in this area. Similarly, these analyses did not consider serially collected disease activity and damage data as the aim of this work was to look at factors at the time of LN onset which predict time to proteinuric remission. Further work involving sophisticated longitudinal modelling of all data would be of interest in future work.

The definition of proteinuric remission used in this study was based on the renal BILAG score. Other cut-offs could have been chosen based on other scoring systems, e.g. the Systemic Lupus Erythematosus Disease Activity Index (SLEDAI) or the European Community Lupus Activity Measure (ECLAM), that both define proteinuric remission as < 0.5 g/24 h, and the ACR score defines it as persistently < 0.5 g/24 h or <+++ on protein dipstix if quantification not performed [28–30]. The BILAG score provides the most comprehensive assessment of renal disease activity of these disease activity scores and includes UPCR or UACR measurements which are more practical in children, less dependent on patient compliance and yield more complete longitudinal data.

The urine samples were collected at different times of the day and in future work it would be useful to standardize the time of sample collection (e.g. all early morning urine samples) to minimize the influence of orthostatic proteinuria. Within the inclusion criteria it was specified that patients must have at least two consecutive follow-up visits with significant proteinuria following its initial proteinuria onset, to minimize the inclusion of patients with one off episodes of orthostatic proteinuria. The study also did not include patients with mild LN within the active LN group (renal BILAG domain score of C) as such a BILAG score can easily be obtained by having 1+ of urine dipstick proteinuria, which may be due to orthostatic proteinuria. This study looked at medication use at the time of LN onset but could not sub-divide patients according to the LN treatment regimen received in view of the multitude of different treatment regimens used in clinical practice over time. The cohort study data is collected alongside routine clinical practice leading to missing data and the need for multiple imputation within the analyses, therefore further analysis in more complete datasets would be of interest in future work.

## Conclusions

A high proportion of JSLE patients develop LN [8, 31, 32]. This study has demonstrated that proteinuria can be persistent within a real world clinical setting. This study has elucidated basic characteristics of patients who are at increased risk of having prolonged proteinuria following an LN flare, namely patients who are younger (< 14 years), have an abnormal eGFR (< 80 mls/min) and concomitant haematological involvement at the time of LN onset. These data cannot guide the clinician as to when to repeat the renal biopsy and/or intensify immunosuppressive treatment, however they do highlight at an early stage, patients who need closer surveillance as they are at increased risk of having a more prolonged period to proteinuric remission. Ideally there would be a specific biomarker or urinary biomarker panel that clinicians could easily test to highlight patients who will display persistence of proteinuria following LN flare [33]. Whilst awaiting this development, it is important to be aware of such clinical and demographic factors. Early reduction in proteinuria following initiation of immunosuppressive therapy has been shown to be associated with improved longer term renal outcomes and survival in adult SLE. Therefore, appreciation of those at risk of prolonged proteinuria may help the clinician to change or fine-tune the intensity and duration of early immunosuppressive therapy.

## Abbreviations

ACR: American College of Rheumatology
anti-dsDNA: anti-double-stranded DNA antibody
BILAG: British Isles Lupus Assessment Grade
C3: complement factor 3
C4: complement factor 4
CI: confidence interval
CRP: c-reactive protein
ECLAM: European Community Lupus Activity Measure
eGFR: estimated glomerular filtration rate
ESR: erythrocyte sedimentation rate
HR: hazard ratio
Ig: immunoglobulin
IVIG: intra-venous immunoglobulin
JSLE: Juvenile Systemic Lupus Erythematosus
LN: lupus nephritis
MMF: mycophenolate mofetil
SLEDAI: Systemic Lupus Erythematosus Disease Activity Index
UACR: urine albumin creatinine ratio
UK: United Kingdom
UPCR: urine protein creatinine ratio
